# miR-31 controls osteoclast formation and bone resorption by targeting RhoA

**DOI:** 10.1186/ar4282

**Published:** 2013-09-03

**Authors:** Fumitaka Mizoguchi, Yousuke Murakami, Tetsuya Saito, Nobuyuki Miyasaka, Hitoshi Kohsaka

**Affiliations:** 1 Department of Medicine and Rheumatology, Graduate School of Medical and Dental Sciences, Tokyo Medical and Dental University, 1-5-45 Yushima, Bunkyo-ku, Tokyo 113-8519, Japan

## Abstract

**Introduction:**

Increased activity of osteoclasts is responsible for bone loss and joint destruction in rheumatoid arthritis. For osteoclast development and bone resorption activity, cytoskeletal organization must be properly regulated. MicroRNAs (miRNAs) are endogenous small noncoding RNAs that suppress expression of their target genes. This study was conducted to identify crucial miRNAs to control osteoclasts.

**Methods:**

miRNA expression in the bone marrow-derived macrophages (BMM) with or without receptor activator of nuclear factor κB ligand (RANKL) stimulation was analyzed by miRNA array. To examine the role of specific miRNAs in osteoclast formation, bone resorption activity and actin ring formation, the BMM were retrovirally transduced with miRNA antagomirs. To confirm whether the suppressive effects on osteoclastogenesis by miR-31 inhibition were mediated by targeting RhoA, osteoclast formation was analyzed in the presence of the RhoA inhibitor, exoenzyme C3.

**Results:**

miR-31 was identified as one of the highly upregulated miRNAs during osteoclast development under RANKL stimulation. Inhibition of miR-31 by specific antagomirs suppressed the RANKL-induced formation of osteoclasts and bone resorption. Phalloidin staining of osteoclasts revealed that actin ring formation at the cell periphery was severely impaired by miR-31 inhibition, and clusters of small ringed podosomes were observed instead. In these osteoclasts, expression of RhoA, one of the miR-31 target genes, was upregulated by miR-31 inhibition in spite of the impaired osteoclastogenesis. Treatment with the RhoA inhibitor, exoenzyme C3, rescued the osteoclastogenesis impaired by miR-31 inhibition.

**Conclusions:**

miR-31 controls cytoskeleton organization in osteoclasts for optimal bone resorption activity by regulating the expression of RhoA.

## Introduction

Osteoclasts, the only cell type capable of resorbing mineralized bone matrix, are derived from hematopoietic precursor cells in the monocyte/macrophage lineage. In collaboration with osteoblasts, osteoclasts play an important role in physiologic bone remodeling to maintain adequate bone volume. In pathological conditions such as osteoporosis and rheumatoid arthritis, increased osteoclastic activity is responsible for bone loss or joint destruction. Understanding the fine tuning of osteoclast activity is important to explain their deregulated functions in pathological conditions.

Cytoskeletal organization is dynamically regulated during osteoclast maturation and bone resorption. Podosomes are adhesion structures consisting of a column of actin filaments surrounded by focal adhesion proteins. During the maturation of osteoclasts, clusters of podosomes assemble into podosome rings, and eventually into a podosome belt. On the mineralized matrix, a large and dense actin ring called the sealing zone is formed. Osteoclasts tightly attach to the bone matrix via the sealing zone to dissolve the mineralized matrix [1]. These processes are regulated by small GTPases, such as RhoA, Rac, CDC42, RhoU and Arf6 [2]. However, how these pathways are regulated during osteoclastogenesis and the bone resorption process is yet to be clarified.

The complex differentiation process of osteoclasts with diverse functions has been explained by a number of molecules and pathways including cytokines, transcription factors, adaptor proteins, kinases and phosphatases [3]. However, additional fine tuning may be required, since differentiation of highly specialized cells is generally thought to be strictly regulated with multiple checkpoints.

MicroRNAs (miRNAs) are noncoding small RNAs about 22 nucleotides in length. By binding to their target sites found mainly in 3'-untranslated regions of multiple genes, miRNAs repress translation of target genes, thereby suppressing the levels of expression. The biological importance of miRNAs has been investigated in several instances, revealing their involvement in fine tuning of many biological processes such as cell proliferation, differentiation and cell death in both physiological and pathological conditions.

We and others have reported the importance of osteoclast miRNAs *in vivo*, and certain miRNAs have been reported to be involved in osteoclast regulation [4-8]. Receptor activator of nuclear factor κB ligand (RANKL) induces expression of miR-21, and its silencing suppresses osteoclastogenesis [6]. miR-155 deficiency suppressed osteoclastogenesis and bone destruction in a murine arthritis model [8]. miR-223 suppressed expression of NFI-A, and inhibition of miR-223 impaired osteoclastogenesis [5]. However, the involvement of these miRNAs in osteoclasts was examined based on previous reports about their expression in other myeloid lineage cells such as monocytes, macrophages and dendritic cells. Here we directly investigated miRNA expression profiles of osteoclasts and identified miR-31 as a crucial miRNA to regulate cytoskeleton organization in these cells.

## Materials and methods

### Osteoclast formation

Bone marrow cells from 6-week-old to 10-week-old DBA1J male mice were cultured in the presence of 20 ng/ml macrophage colony-stimulating factor (M-CSF; R&D Systems, Minneapolis, MN, USA) for 2 days to induce bone marrow macrophages (BMMs). BMMs were plated at 1.5 × 10^3 ^cells/cm^2^, and were cultured in the presence of 20 ng/ml M-CSF and 5 to 50 ng/ml RANKL (R&D Systems) for 72 to 120 hours to induce osteoclasts. Animal experiments were approved by the animal welfare committee of Tokyo Medical and Dental University.

### miRNA array analysis

BMMs were cultured with 20 ng/ml M-CSF in the presence or absence of 50 ng/ml RANKL for 24 hours, and were lysed for total RNA isolation. Cy3-labeled RNA from control BMMs and Cy5-labeled RNA from RANKL-treated BMMs were mixed and hybridized to the miRNA array using the MiRVana™ MiRNA Bioarray system Ver. 2 (Ambion, Austin, TX, USA). Data were analyzed by microarray data analysis tool software (Filgen, Nagoya, Japan). The data have been deposited in the NCBI Gene Expression Omnibus [GEO:GSE48629].

### Reverse transcription and real-time quantitative PCR

For the real-time quantitative PCR analysis of miR-31, Taq Man Gene Expression Assays (Applied Biosystems, Foster City, CA, USA) were used. PCR was conducted according to the manufacturer's protocol. The expression levels of miR-31 were evaluated by calculating the ratios against U6B expression levels.

### Retroviral transduction

A miR-31-specific inhibitory oligonucleotide was cloned into retroviral vector, pSilencer5.1-H1Retro (Ambion). Sequences of the miR-31 inhibitory oligonucleotide were as follows: sense, 5'-GATCCGAGCTATGCCAGCATCTTGCCTTTTTTTGGAAA-3'; anti-sense, 5'-AGCTTTTCCAAAAAAAGGCAAGATGCTGGCATAGCTCG-3'. Bone marrow cells were cultured in the presence of 50 ng/ml M-CSF for 24 hours to induce BMMs. BMMs were infected with miR-31 silencing (pSmiR31) or control (pScontrol) virus for 6 hours, and cultured in the presence of 6 μg/ml puromycin for 2 days. pMX-NFATc1 was provided by Dr Hiroshi Takayanagi (Tokyo University, Tokyo, Japan) and has been described previously [9].

### Pit formation assay

BMMs were cultured on Osteologic (BD Biosciences, Bedford, MA, USA) in the presence of 20 ng/ml M-CSF and 5 to 20 ng/ml RANKL for 6 days. Resorption lacunae were visualized by dark-field light microscopy for analysis with Image J software (NIH, Bethesda, MD, USA).

### Actin ring staining

After fixation, cells were incubated in 0.1% Triton X-100 in PBS for 5 minutes, and stained by Alexa Fluor 488 phalloidin (Molecular Probes, Eugene, OR, USA).

### Western blot analysis

Total cell lysates were subjected to western blot analysis with specific antibodies against RhoA (clone 67B7; Cell Signaling, Danvers, MA, USA), c-Src (clone N-16; Santa Cruz, Dallas, TX, USA), Pyk2 (clone H364; Cell Signaling) and beta-actin (clone AC-15; Sigma Aldrich, St Louis, MO, USA) together with peroxidase-conjugated anti-rabbit or anti-mouse IgG as secondary antibodies.

### RhoA activity assay

GTP-bound active RhoA was quantified using the G-LISA RhoA activation assay biochem kit (Cytoskeleton, Denver, CO, USA) according to the manufacturer's protocol.

### Pharmacological RhoA inhibition

For the pharmacological RhoA inhibition, cell-permeable exoenzyme C3 transferase (Cytoskeleton) was used. After culturing BMMs in the presence of 20 ng/ml M-CSF and 5 to 20 ng/ml RANKL for 24 hours, they were further cultured in cell-permeable exoenzyme C3 containing medium for 48 hours. After tartrate-resistant acid phosphatase (TRAP) staining, TRAP-positive multinucleated cells were counted as osteoclasts.

### Statistical evaluations

All of the numeral data in Results are presented as mean ± standard deviation. Statistical analysis was performed based on Student's *t *test or Welch's *t *test based on the results of the *F *test.

## Results

### miR-31 is one of the highly upregulated miRNAs in osteoclasts

**Figure 1:**
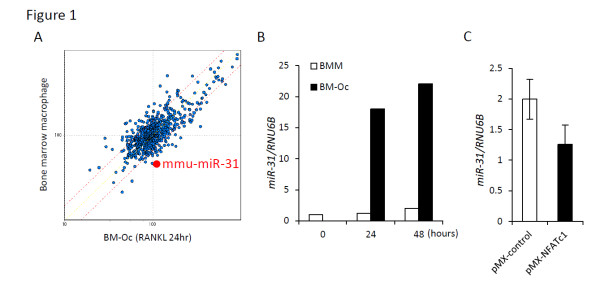
**miR-31 is one of the highly upregulated microRNAs during osteoclastogenesis**. **(A) **The profile of microRNA (miRNA) expression levels was visualized by a scatter plot of signal intensities. Horizontal axis, cells treated with macrophage colony-stimulating factor (M-CSF) and receptor activator of nuclear factor κB ligand (RANKL) for 24 hours (BM-Oc); vertical axis, cells treated only with M-CSF (bone marrow macrophage (BMM)). Dashed red lines indicate 1.5-fold upregulated or downregulated point, and miR-31 is indicated as a red dot. **(B) **Expression of miR-31 during osteoclastogenesis was examined by real-time quantitative PCR at the indicated time points. Values were normalized against U6B. RANKL treatment of BMMs (BM-Oc) enhanced the expression of miR-31. **(C) **Real-time quantitative PCR analysis of miR-31 after the transduction with retroviruses encoding NFATc1 (pMX-NFATc1) or control viruses (pMX-control). Overexpression of NFATc1 did not increase the level of miR-31. Nfatc1, nuclear factor of activated T-cells, cytoplasmic, calcineurin-dependent 1.

To identify the miRNAs that are involved in osteoclastogenesis, we conducted a miRNA array and found 31 upregulated miRNAs and 11 downregulated miRNAs (>1.5-fold) following RANKL stimulation (Figure 1A). Of these miRNAs, we focused our analysis on one of the highly upregulated miRNA, miR-31. In real-time quantitative PCR analysis, expression of miR-31 in BMM was low. However, RANKL treatment enhanced miR-31 expression up to 18-fold (Figure 1B).

To examine whether the increased level of miR-31 during osteoclastogenesis is mediated by nuclear factor of activated T-cells, cytoplasmic, calcineurin-dependent 1 (Nfatc1), a master transcription factor in osteoclastogenesis, Nfatc1 was retrovirally overexpressed in BMMs. In real-time quantitative PCR analysis, overexpression of Nfatc1 increased the level of Nfatc1 by about 20-fold, compared with the empty vector control. However, expression levels of miR-31 were not increased by the overexpression of Nfatc1, indicating that the expression of miR-31 is not regulated by Nfatc1 (Figure 1C).

### Inhibition of miR-31 suppresses osteoclastogenesis and bone resorption

**Figure 2:**
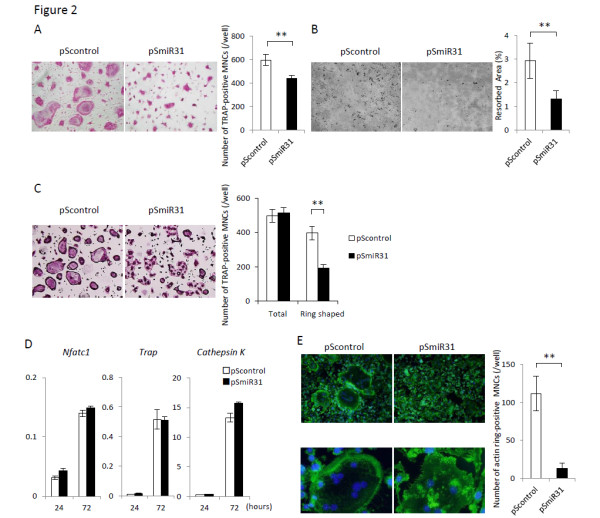
**Osteoclasts generated under miR-31 inhibition show impaired osteoclastogenesis, bone resorption activities and actin ring formation**. **(A) **Tartrate-resistant acid phosphatase (TRAP) staining of osteoclasts after transduction with retroviruses encoding antagomirs of miR-31 (pSmiR31) or control viruses (pScontrol). Osteoclasts were developed in the presence of receptor activator of nuclear factor κB ligand (RANKL) for 72 hours. Under conditions of miR-31 inhibition, although TRAP-positive multinucleated cells (MNCs) were formed, the frequency of ring-shaped mature osteoclastic cells was decreased. The number of TRAP-positive MNCs was decreased by miR-31 inhibition (*n *= 5/group). **(B) **Pit formation assay. Resorption lacunae were visualized in black by dark-field light microscopy. The resorbed area was decreased by miR-31 inhibition (*n *= 3/group). **(C) **TRAP staining of osteoclasts that were developed in the presence of RANKL for 120 hours. The total number of TRAP-positive MNCs that were generated under miR-31 inhibition was equivalent to that of control. The number of ring-shaped TRAP-positive MNCs that were generated under miR-31 inhibition was less than that of control (*n *= 5/group). **(D) **Real-time PCR analysis of osteoclasts generated under miR-31 inhibition. Values were normalized to glyceraldehyde 3-phosphate dehydrogenase expression. Expression levels of Nfatc1, Trap and Cathepsin K were not altered by miR-31 inhibitions (*n *= 3/group). **(E) **Actin-ring staining of osteoclasts. The number of actin ring-positive MNCs was decreased by miR-31 inhibition (upper panels) (*n *= 3/group). Podosome organization at the cell periphery was observed in control osteoclasts (lower left panel), but not when miR-31 was inhibited. Instead, small ring-shaped podosomes were observed (lower right panel). Green, Alexa 488 phalloidin; blue, 4',6-diamidino-2-phenylindole. **P < 0.01. Nfatc1, nuclear factor of activated T-cells, cytoplasmic, calcineurin-dependent 1.

To examine the role of miR-31 in osteoclastogenesis, BMMs were treated with miR-31 antagomirs, which inhibit binding of miR-31 to its target mRNAs. This antagomir did not alter the expression levels of other miRNAs, including miR-21, miR-155 and miR-223, which have been reported to be involved in the regulation of osteoclast (see Additional file 1). Under control conditions without the antagomir treatment, ring-shaped TRAP-positive multinucleated osteoclastic cells were observed after promotion of osteoclast formation with RANKL for 72 hours. By contrast, when miR-31 was inhibited, the number of TRAP-positive multinucleated cells was significantly decreased, and ring-shaped mature osteoclastic cells were rarely observed (Figure 2A). Osteoclasts generated under miR-31 inhibition also resorbed a synthetic calcium phosphate matrix less efficiently than normal osteoclasts (Figure 2B).

To examine whether decreased osteoclast formation via miR-31 inhibition was caused by the impairment of the fusion process, we cultured osteoclasts for 120 hours and the number of TRAP-positive multinucleated cells was evaluated. Following a period of these culture conditions, the total number of TRAP-positive multinucleated cells with miR-31 inhibition was equivalent to that of the control (Figure 2C). However, the number of ring-shaped TRAP-positive multinucleated cells with miR-31 inhibition was still less than the control (Figure 2C). These results indicate that miR-31 does not affect the fusion process.

To study the pathways of miR-31 effects on osteoclastogenesis and bone resorption activities, real-time PCR was conducted to analyze the expression of genes related to osteoclast formation and maturation. Inhibition of miR-31 had no effect on Nfatc1, the master regulator of osteoclastogenesis, or on Trap or Cathepsin K (Figure 2D). These results suggest that suppression of osteoclastogenesis and bone resorption activity were attributable to inhibition at the late stages of osteoclastogenesis.

### Osteoclasts generated under miR-31 inhibition have impaired actin ring formation

The sealing zone has to be formed for bone resorption by osteoclasts. Since this formation is mediated by the organization of the actin cytoskeleton called the actin ring, we examined actin ring formation in osteoclasts developed under miR-31 inhibition. Without miR-31 inhibition, actin fibers accumulated in the periphery of the osteoclast cytoplasm (Figure 2E, upper left panel). However, when miR-31 was inhibited, peripheral actin rings were rarely formed (Figure 2E, upper right panel). At high magnification, the shape of the control-treated osteoclasts was round and the edge of the cell was smooth, accompanying the accumulation of actin fibers (Figure 2E, lower left panel). By contrast, the shape of the miR-31-inhibited osteoclasts was not round, but instead had an irregular edge. Instead of actin rings in the periphery, ring-shaped small clusters of actin were observed in the center of the cells (Figure 2E, lower right panel). These results suggest that impaired osteoclast formation and bone resorption resulting from miR-31 inhibition are attributable to cytoskeleton disorganization.

### Osteoclasts generated under miR-31 inhibition show increased RhoA activity

**Figure 3:**
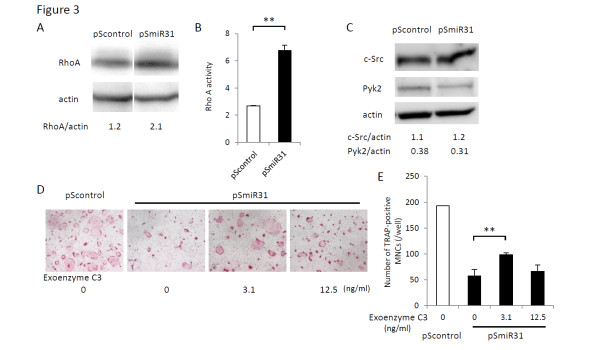
**Impaired osteoclastogenesis by miR-31 inhibition is mediated by increased RhoA activity**. **(A) **Western blot analysis of osteoclasts after retroviral transduction of pSmiR31 or pScontrol. Expression of RhoA protein was increased in pSmiR31 transduced osteoclasts. **(B) **RhoA activity was also increased by miR-31 inhibition. Values were normalized to RhoA activity of bone marrow macrophages (BMMs) (*n *= 3/group. **P < 0.01. **(C) **Western blot analysis of osteoclasts. Expression of c-Src and Pyk2 protein were not altered in pSmiR31 transduced osteoclasts. **(D), (E) **Osteoclasts were induced in the presence of the RhoA inhibitor; exoenzyme C3. A low concentration of exoenzyme C3 rescued the decreased number of ring-shaped tartrate-resistant acid phosphatase (TRAP)-positive MNCs caused by miR-31 inhibition (*n *= 3/group). **P < 0.01. MNCs, multinucleated cells.

We assumed that miR-31 should target the genes that regulate cytoskeleton organization. Among them, we focused on one of the GTPase, RhoA. RhoA has been reported to be one of the targets of miR-31 in breast cancer [10], and plays an important role as a molecular switch in transducing extracellular signals to actin and microtubule cytoskeleton [11]. In fact, osteoclasts with miR-31 inhibition expressed increased levels of RhoA protein (Figure 3A). GTP-bound active RhoA was also increased by miR-31 inhibition, in spite of impaired osteoclast formation (Figure 3B). In contrast, c-Src and Pyk2 expression levels were not altered by miR-31 inhibition (Figure 3C). These results argue that RhoA is a target of miR-31 in osteoclasts.

### Impaired osteoclastogenesis by miR-31 inhibition is rescued by pharmacological RhoA inhibition

To determine whether the suppressive effect of miR-31 inhibition on osteoclasts could be mediated by increased expression of RhoA, BMMs treated with the miR-31 antagomir were induced to osteoclasts in the presence of the RhoA inhibitor, exoenzyme C3. A low concentration of exoenzyme C3 rescued the decreased number of ring-shaped TRAP-positive multinucleated cells caused by miR-31 inhibition (Figure 3D, E). This result indicates that impaired osteoclastogenesis caused by miR-31 inhibition is due to excessive RhoA expression.

## Discussion

In this study, we identified miR-31 as an important miRNA in osteoclast regulation. Several miRNAs are known to be involved in the regulation of osteoclasts [5,6,8]. Unlike these other miRNAs, however, miR-31 has not been reported to have critical roles in other hematopoietic myeloid lineage cells. Expression of miR-31 was induced by RANKL, and it controlled osteoclastogenesis and bone resorption by regulating the cytoskeleton organization.

Our results indicate that the main effect of miR-31 inhibition is to suppress the function of osteoclasts, since bone resorption and actin ring formation were severely suppressed by miR-31 inhibition in spite of the normal expression of Nfatc1, Trap and Cathepsin K. In addition, since osteoclast formation was also suppressed by miR-31 inhibition, miR-31 might be involved in the differentiation process as well, although the level was moderate. We observed that miR-31 regulates cytoskeletal organization in osteoclasts. Cytoskeletal organization is important in both osteoclast differentiation and the resorption processes. Since expression level of miR-31 was increased during the osteoclast formation, miR-31 plays a role in the late phase of differentiation and the resorption activity.

RhoA was identified as a miR-31 target gene in osteoclasts, where it regulates formation of actin stress fibers and focal adhesions [12]. Inhibition of RhoA by exoenzyme C3 has been reported to inhibit both actin ring formation and pit formation on dentine slices [13]. Furthermore, transduction of active RhoA promoted actin ring formation [14]. These results indicate that activation of RhoA is essential for actin ring formation in osteoclasts. On the other hand, inhibition of RhoA in the early stages of osteoclast differentiation increased the formation of actin ring-positive mature osteoclasts [15]. Moreover, Pyk2-deficient osteoclasts showed impaired sealing zone formation and bone resorption via enhanced RhoA activity [16]. These results demonstrated that excessive activity of RhoA also suppresses osteoclastogenesis and sealing zone formation, indicating that RhoA activity must be regulated tightly during osteoclast formation and bone resorption.

During the process of osteoclastogenesis and bone resorption, a regulator of RhoA expression must exist. However, such RhoA regulatory systems in osteoclasts had not yet been clarified. We observed that RhoA activities in osteoclasts were higher than in BMMs (data not shown), and that expression of miR-31 was also increased during osoteoclastogenesis. Therefore, we hypothesized that miR-31 plays an important role in regulating the expression of RhoA at an appropriate level to control osteoclastogenesis and bone resorption.

The ability of exoenzyme C3 to rescue the impaired osteoclastogenesis by miR-31 inhibition confirmed that RhoA is a major target gene of miR-31 in osteoclasts. However, its effect was partial, suggesting that miRNA pleiotropically regulates the expression of multiple genes. Other miR-31 target genes might also control osteoclast development and function.

Our miRNA array analysis of osteoclasts revealed that the miRNA expression profile was changed after stimulation with RANKL. During osteoclastogenesis the gene expression profile also changes, and this must be tightly regulated. miRNA is one such regulatory system that we have shown is involved in gene expression in osteoclasts.

## Conclusions

miR-31 is one of the crucial molecules to finely control the development and function of osteoclasts. Excessive and insufficient activity of RhoA both suppressed osteoclastogenesis. Suppression by miR-31 should optimize the expression of RhoA involved in cytoskeletal organization and following bone resorption.

## Abbreviations

BMM: bone marrow macrophage; M-CSF: macrophage colony-stimulating factor; miRNA: microRNA; nfatc1: nuclear factor of activated T-cells: cytoplasmic: calcineurin-dependent 1; PCR: polymerase chain reaction; RANKL: receptor activator of nuclear factor κB ligand; TRAP: tartrate-resistant acid phosphatase.

## Competing interests

This work was supported by grant from Ministry of Education, Culture, Sports, Science and Technology in Japan (21249060, 21790944, Global Center of Excellent program, International Research Center for Molecular Science in Tooth and Bone diseases) and Lilly Grant Office.

## Authors' contributions

FM participated in the design of the study, carried out the experiments, and drafted the manuscript. YM participated in the design of the study and carried out the experiments. TS carried out the experiments. HK and NM participated in study design and coordination, and helped to draft the manuscript. All authors read and approved the final manuscript.

## Supplementary Material

Additional file 1 **Figure S1 showing real-time PCR analysis of osteoclasts generated under miR-31 inhibition**. Values were normalized to U6B expression. Expression levels of miR-21, miR-155 and miR-223 were not altered by miR-31 inhibitions (*n *= 3/group). Click here for file
