# Supplementary figures and images for: miR-31 controls osteoclast formation and bone resorption by targeting RhoA
Source: Arthritis Res Ther. 2013 Sep 3;15(5):R102. doi: 10.1186/ar4282 (PMC3978447; doi:10.1186/ar4282)

Figure S1

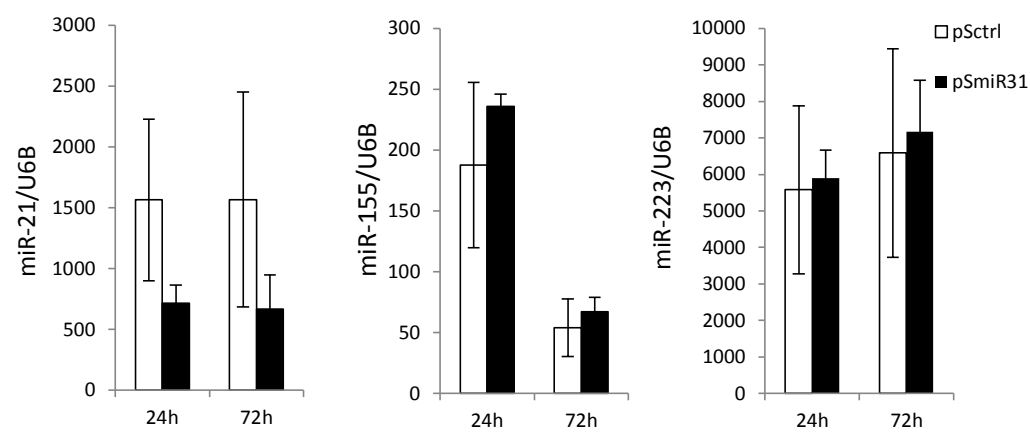

Supplement: Additional file 1 — Figure S1 showing real-time PCR analysis of osteoclasts generated under miR-31 inhibition. Values were normalized to U6B expression. Expression levels of miR-21, miR-155 and miR-223 were not altered by miR-31 inhibitions (n = 3/group). [file ar4282-S1.PDF]
